# Academic Promotion of Physicians in Medical Schools: A Special Focus on Primary Health Care in Taiwan

**DOI:** 10.3390/ijerph18189615

**Published:** 2021-09-12

**Authors:** Hsin Ma, Feng-Yuan Chu, Tzeng-Ji Chen, Shinn-Jang Hwang

**Affiliations:** 1 Department of Family Medicine, Taipei Veterans General Hospital, Taipei 112, Taiwan; tov8588@gmail.com (H.M.); sjhwang@vghtpe.gov.tw (S.-J.H.); 2 Division of Clinical Toxicology and Occupational Medicine, Department of Medicine, Taipei Veterans General Hospital, Taipei 112, Taiwan; steven2259898@gmail.com; 3 School of Medicine, National Yang Ming Chiao Tung University, Taipei 112, Taiwan; 4 Big Data Center, Department of Medical Research, Taipei Veterans General Hospital, Taipei 112, Taiwan

**Keywords:** career mobility, journal impact factor, medical faculty, primary health care, publishing

## Abstract

The quality and quantity of papers published in journals play a crucial role in achieving an academic promotion in medical schools. Reports on the criteria for promotion and their impact on different specialties, especially on primary health care, which has low research output, are rare. We investigated the scoring systems generally adopted for academic promotion at most medical schools in Taiwan. The weighted scores were derived from the multiplication of weights from categories of paper, journal impact factor, or ranking in a certain category by impact factor, and author order. To determine the thresholds of papers required for different levels of promotion, we took papers in the highest- or lowest-ranked journals in the primary health care category in 2019 Journal Citation Reports as examples. Considering publications in the highest-ranked journals, a median of 4.6 first or corresponding author papers were required for a professorship, as well as 3.3 for an associate professorship, and 2.5 for an assistant professorship. In contrast, a median of 30, 20, and 13.5 papers in the lowest-ranked journals was required for the corresponding positions. Thus, academic promotions for primary health care educators in Taiwan are highly demanding. The detrimental effects of scoring systems deserve further research.

## 1. Introduction

Clinician educators, who work as skilled physicians and teachers, play an indispensable role in medical institutions [1,2] and face academic promotion in medical schools. According to previous studies related to academic promotion, in a radiology department, clinician educators need to be aware of promotion criteria [3]. Assessment of requirements for promotion in dental schools has been extensively reported. The complexity of promotion policies and the significance each university allocates to research varies [4,5]. The clinical service, teaching, and publications were evaluated in academic promotion of psychiatrists [6]. In a study involving orthopedic surgeons in the United States, the scholastic productivity index impacted academic rank [7]. Recently, a study surveyed family medicine department chairs in the United States and found that clinic demands may reduce research engagement and consequently the publications, which are important for academic promotion [8].

The number of publications, journal impact factor, and authorship order were key elements in promotion criteria and scoring systems in medical schools [9,10]. Nevertheless, based on the 2019 Edition of InCites Journal Citation Reports (JCR), such regulations pose an obstacle in the promotion of primary health care educators [11]. The impact factor of the top-ranked journal in primary health care was 4.686 which is relatively low compared to that in other disciplines. Besides, the number of articles published in primary health care is comparatively small, merely 1458 papers in 19 journals in 2019. In contrast, 7475 papers were published in 68 dermatology journals, and 19,785 papers in 138 journals of cardiac and cardiovascular systems. Primary health care educators seem to face difficulties in obtaining academic promotion because a fewer number of papers related to primary health care are published and journals in primary health care have lower impact factors in JCR. However, the difficulty encountered in achieving academic promotion by primary health care educators owing to the scoring systems has been rarely reported.

In this study, we tried to analyze the scoring systems for academic promotion at different medical schools in Taiwan and identify the obstacles that primary health care educators dealt with under these systems. Few studies have described the scoring systems in such depth, and it is particularly stringent for specialties with low impact factors, such as primary health care. The findings of our study will provide thorough information for primary health care educators worldwide who face similar challenges in academic promotion. Furthermore, our study may serve as an international reference to avoid the adoption of such scoring systems in academic promotion.

## 2. Materials and Methods

### 2.1. Background

#### 2.1.1. A Brief History of Medicine in Taiwan

Taiwan is located in East Asia and had a population of 23,561,236 by the end of 2020 [12]. Medical missionaries first introduced Western medicine to Taiwan during the mid-nineteenth century. The first medical school in Taiwan was established in 1899 when Taiwan was a dependency of Japan, which led to the modernization of the medicine and healthcare systems in Taiwan [13]. To date, 13 medical schools have been established, four of which are public schools, and one is a military school. Geographically, seven medical schools are in northern Taiwan, two in central Taiwan, three in southern Taiwan, and one in eastern Taiwan. Approximately 1500 medical school students graduate per year [14] and receive residency programs [15]. As of 2019, Taiwan has 49,791 practicing physicians according to the medical statistics of the Taiwan Medical Association [14]. Of 23 different specialties in Taiwan, family medicine was officially established in 1988, with nearly 4000 certified specialists, forming the third-largest medical association until 2019.

#### 2.1.2. Family Medicine Teachers in Taiwan

Adapted from the 2019 statistics of the Taiwan Association of Family Medicine [16], 116 full-time and adjunct staff were certified by the Ministry of Education as family medicine teachers at 13 medical schools in Taiwan: professors (10.3%), associate professors (16.4%), assistant professors (31.0%), and lecturers (42.2%) (Table 1).

#### 2.1.3. The Process of Examining Qualifications for Academic Promotion in Taiwan

According to the Act Governing the Appointment of Educators [17], the Ministry of Education in Taiwan imposes minimum general criteria for promotion, requiring years of service and specialized publications, without mentioning the minimum number of International Scientific Indexing articles and the order of authors. The eligibility of a clinician educator is checked through a step-by-step process that is similar across all the 13 medical schools in Taiwan. When a physician applies for promotion, the department first conducts a preliminary review of qualifications, which is followed by a second review by the Personnel Office before an external review. After passing the external review, the results of the external review and the application information are submitted to the faculty evaluation committee for final assessment. Once approved, the Personnel Office submits the list of promoted teachers to the Ministry of Education for certification.

#### 2.1.4. Scoring System of Academic Promotion in Taiwan

In Taiwan, scoring systems have been adopted to quantify academic performance and promotion criteria for university faculty members at different levels. Table 2 shows the scoring system at the medical school of National Yang Ming Chiao Tung University (NYCU). The weighted score of an academic publication is calculated based on the weights from the category of paper (C), the ranking of the Science Citation Index (SCI), the journal publishing the paper (J) in a certain category, and the author order (A). C is multiplied by J and then by A (C × J × A) to obtain the weighted score of a paper. The weighted score of each paper published by a clinician educator is added together to obtain the total score. The total score should be greater than a certain threshold for promotion (e.g., a total score greater than 500 is required for an associate professor to be promoted to a professor in NYCU).

### 2.2. Data Collection

The list of regulations for academic promotion in Taiwan was collected by individually entering the name of each medical school into the Google search engine. Then, the regulations of academic promotion posted on the official website of each medical school were downloaded for analysis on 21 March 2021. All 13 medical schools in Taiwan including National Taiwan University (NTU), NYCU, National Defense Medical Center (NDMC), Taipei Medical University (TMU), Fu Jen Catholic University (FJU), Mackay Medical College (MMC), Chang Gung University (CGU), China Medical University(CMU), Chung Shan Medical University (CSMU), National Cheng Kung University (NCKU), Kaohsiung Medical University (KMU), I-Shou University (ISU), and Tzu Chi University (TCU) were included in this study (Supplementary Data Table S1). The required data for analysis were retrieved by browsing through the relevant regulations.

### 2.3. Study Design

#### 2.3.1. Scoring System of Medical Schools in Taiwan

In Taiwan, though other factors are affecting academic promotion, it is mainly allowed after the score calculated by the sum of the multiplication of (C × J × A) passes a certain threshold. In addition to this predominant scoring system, some medical schools follow the research performance index (RPI). RPI has been established by the Biological Sciences Development Division of the National Science Council as one of the indicators of research performance for assessing the accomplishments of researchers in Taiwan.

We explored the scoring system of each medical school in Taiwan which adopted weighted scores composed of the weights from the category of paper (C), the impact factor or the ranking of the impact factor in a certain category (J), and the author order (A). We described the weights of an original article, an impact factor according to its value or ranking in a certain category, and for the first author. Considering the ranking of an impact factor, only the weights of the ranking less than or equal to 10% and the ranking greater than 80% were present. Most medical schools adopted threshold scores for application, except NTU and NDMC, which used a full score system. However, a full score system implies that most applicants reach the goal, and those who do not would be eliminated. Thus, the full scores were equally deemed as thresholds for applicants. CMU uses RPI as a threshold, which needed to be reached after a score was calculated according to a sum of the multiplication of (C × J × A) and an anticipated number of papers. CGU uses a sum of the number of papers or a sum of impact factors from papers as a threshold instead of a total score obtained by adding each weighted score calculated by (C × J × A) from each paper.

Due to different thresholds and scoring methods of the 13 medical schools, we calculated the number of papers equivalent to different promotion thresholds required at each medical school. *Annals of Family Medicine* ranked 1st in the 2019 SCI database among 19 journals indexed under the “primary health care” category [11]. The impact factor of *Annals of Family Medicine* was 4.686 and was used to calculate the numbers of equivalent papers. On the contrary, the *Journal of Family Practice* ranked 19th. The impact factor of the *Journal of Family Practice* was 0.694 and was also used to calculate the numbers of equivalent papers.

Most medical schools employed threshold scores for promotion criteria of professor, associate professor, and assistant professor. The qualification for the selection of a lecturer was excluded in our study as there were no explicit scoring regulations for lecturers in several medical schools.

#### 2.3.2. The Impacts of Scoring System on Different Medical Specialties

The NYCU School of Medicine website indicated that it consists of 27 departments [18]. Twenty of the departments were managed by 20 different medical specialties and therefore the 20 specialties were presented in the study. In addition, according to the training program from the Taiwan Society of Internal Medicine [19] and the subdivisions of the department of internal medicine in Taipei Veterans General Hospital, one of the 20 specialties, internal medicine, was further divided into nine subspecialties. In JCR, we searched for different journal categories corresponding to the specialties and subspecialties. The impact factor of the top ranked journal in each category was listed and used to calculate the number of papers equivalent to different promotion thresholds in respective specialty or subspecialty. This revealed the different levels of merit required by these medical specialties and subspecialties in offering an academic promotion.

#### 2.3.3. Other Information from Regulations of Academic Promotion

We also searched for other criteria, such as a higher weight from a paper published in a journal with an extraordinarily high-impact factor or ranking, or weight from a paper published in a non-SCIE/SSCI or a domestic journal. Other promotion tracts including excellent teaching abilities and contribution in medicine-related industries were also studied. We also searched for regulations for counting the maximum number of publications for a professor, associate professor, and assistant professor.

### 2.4. Statistical Analysis

Descriptive statistics in Microsoft Excel 2016 were used to display the numeric results. The box plot was created by R version 4.0.5 [20].

### 2.5. Ethical Approval

According to the legislation of personal data privacy in Taiwan and regulations of the institutional review board at Taipei Veterans General Hospital, the use of publicly available data is exempted from approval.

## 3. Results

### 3.1. Comparison of Different Weights Conditional on an Original Article Published by a Clinician-Educator as the First Author in Different Schools

The weighted score (C) for an original article in all 13 schools was 3. If the impact factor ranking was less than 5%, the weighted score (J) was 10 for TCU, 8 for NYCU, and NCKU, 7.5 for ISU, 5 for FJU, and 6 for the rest of the schools. As for the first author (A), only NDMC has a weighted score of 6, while all others have a weighted score of 5 (Table 3).

### 3.2. Comparison of Academic Promotion Scoring Systems in Different Schools Using the Numbers of Papers Equivalent to Different Thresholds

#### 3.2.1. Equivalent Number of Papers Calculated Using the Impact Factor of the Top-Ranked Journal as a Weight

**Figure 1:**
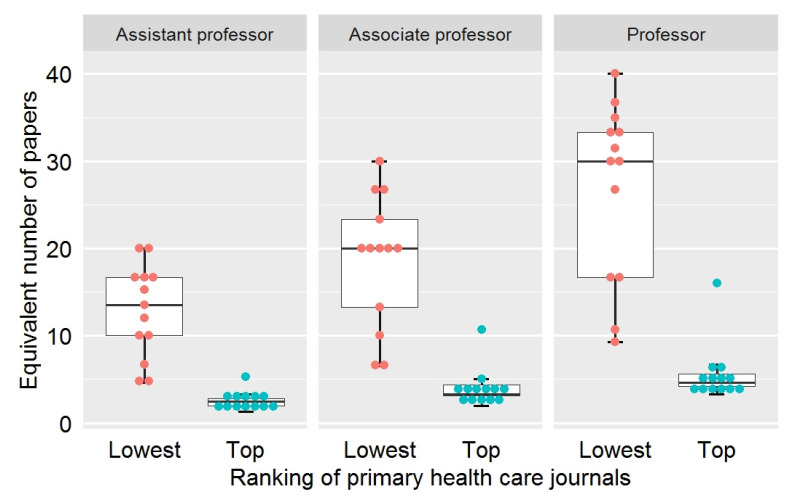
The distribution of the numbers of papers equivalent to different promotion thresholds using the impact factor of the top-ranked journal or that of the lowest-ranked journal in primary health care as a weight.

Among 13 medical schools, applicants required a minimum of 3.3, a maximum of 16, and a median of 4.6 equivalent of papers in the top journals of primary health care for promotion from associate professors to professors (Table 4, Figure 1).

As for promotion from assistant professors to associate professors, applicants required a minimum of 2, a maximum of 10.7, and a median of 3.3 equivalent of papers in top-tier journals. A minimum of 1.6, a maximum of 5.3, and a median of 2.5 equivalent of papers in the top-ranked journals were essential for lecturers to be promoted to assistant professors.

#### 3.2.2. Equivalent Number of Papers Calculated Using the Impact Factor of the Lowest-Ranked Journal as a Weight

Among 13 medical schools, eligibility for promotion from associate professors to professors required a minimum of 9.3 and a maximum of 36.7 papers, with a median of 30 papers. For the promotion of assistant professors to associate professors, at least 6.5 papers, up to 30 papers, and a median of 20 papers was needed. For the promotion of lecturers to assistant professors, the minimum number of papers was 4.6, the maximum was 20, and the median was 13.5 papers.

### 3.3. Maximum of Papers Counted

Six schools had regulations for counting a maximum number of papers (Table 5). NTU counted a maximum of 12 papers in the applications for professors, assistant professors, and associate professors. TMU, MMC, NCKU, and KMU counted a maximum of 15 papers for applications for professors, assistant professors, and associate professors. NYCU counted a maximum of 12 papers from associate professors to professors, 10 papers from assistant professors to associate professors, and 8 papers from lecturers to assistant professors.

### 3.4. Bonus Weights of Papers Published in High-Impact-Factor Journals

Some medical schools set the weight of a journal (J) not only according to the rank of a journal impact factor but also the impact factor itself, especially when the impact factor was thought to be high. A higher weight would be used after a comparison between weight from ranking and that from impact factor itself.

In NTU, if a journal’s impact factor is larger than or equal to five, the impact factor itself and weight according to the journal’s ranking would be compared. The larger one would be used as a final weight from the journal. In NDMC, if the ranking of a journal is less than or equal to 20%, the weight is assumed to be 6. In NYCU, if a journal’s impact factor is larger than 5 or ranking less than or equal to 10%, the weight would be equal to the impact factor (if the impact factor is more than 8) or 8. However, if an impact factor is larger than 10, the weight might be equal to 1.5 times the impact factor. In TMU, if a journal’s 5-year impact factor is larger than or equal to 6, the 5-year impact factor itself and weight according to the journal’s ranking would be compared. The larger one would be used as a final weight from a journal. In FJU, MMC, CMU, and CSMU, if a journal’s impact factor is larger than or equal to 6, the impact factor itself and weight according to the journal’s ranking would be compared. The larger one would be used as a final weight from a journal. In NCKU, if a journal’s impact factor is greater than 5 and less than or equal to 10, the weight would be equal to the impact factor. If a journal’s impact factor is greater than 10, the weight would be equal to 1.5 times the impact factor. In KMU, if a journal’s impact factor is larger than five, the impact factor itself and weight according to the journal’s ranking would be compared. The larger one will be used as a final weight from a journal. In ISU, if a journal’s impact factor is greater than or equal to 10, the weight would be 15. If a journal ranking is less than 10% or a journal’s impact factor is greater than or equal to 5 and less than 10, the weight would be 7.5. If a journal ranking is greater or equal to 10% and less than 20%, or a journal’s impact factor is greater or equal to 2 and less than 5, the weight would be 5. In TCU, if a journal’s impact factor is greater than or equal to 10, the weight would be equal to the impact factor. If a journal has a ranking less than 5% or an impact factor greater than or equal to 5, the weight would be 10. If a journal has a ranking greater than or equal to 5 and less than 10%, or an impact factor greater than or equal to 3.5 and less than 5, the weight would be 8. If a journal has a ranking greater or equal to 10 and less than 25%, or an impact factor greater than or equal to 2 and less than 3.5, the weight would be 6.

### 3.5. Inclusion of Papers in Non-SCIE/SSCI Journals and Domestic Journals

All medical schools in Taiwan included non-SCIE/SSCI papers in their scoring system of academic promotion. Although non-SCIE/SSCI papers were considered in grading, their weights were lower compared to SCIE/SSCI papers. For instance, to achieve the same target weight at NYCU, one publication in a top-most primary health care journal is tantamount to publishing four papers in non-SCIE/SSCI journals. Most medical schools encouraged authors to submit to domestic journals, such as *Taiwan Journal of Public Health* and *Journal of Medical Education*, except NTU and FJU. Papers in domestic journals are also counted for weighted scores though their weights are lower compared to the papers in SCIE/SSCI journals.

### 3.6. Diverse Promotion Tracks

Other tracks for promotion included excellent teaching abilities and contributions in medicine-related industries. To encourage teachers with outstanding teaching performance and important teaching tasks/skills, all medical schools provide a teaching track for educators primarily engaged in teaching and require a lower thesis threshold. A contributor in medicine-related industries can gain points based on technology transfer, industry-academia cooperation, patent certification, derivative start-ups, innovation awards, and research results.

### 3.7. Bonus Points for Additional Items

One of the medical schools not only adopted journal impact factor, but also article-based citation measure as bonus points. The H-index is considered as an additional item in KMU. The reference values of H-index are 15 for professor, 10 for associate professor, and 5 for assistant professor, respectively. If an applicant has an H-index above the reference value, one extra unit of H-index will earn her/him 0.5 point in weighted score. A maximum of 10 points can be gained from H-index. Besides, books are not considered in calculating scores in all medical schools.

### 3.8. List of Top-Ranked Journal of Medical Specialties and Subspecialties

Table 6 lists the top-ranked journals in medical specialties and subspecialties in JCR. The impact factors of the top journals in all medical specialties except otolaryngology (impact factor = 3.848) are higher than those of family medicine (impact factor = 4.686). If a gastroenterologist wants to be promoted from an associate professor to a professor in NYCU, he or she solely needs to publish one original paper in Nature Reviews Gastroenterology & Hepatology (impact factor = 29.848) as the first author. The total score would be calculated as 3 × 29.848 × 1.5 × 5 = 671.58, which is well above the threshold score for promotion.

## 4. Discussion

The present study investigated academic scoring systems in different medical schools and the challenges that primary health care educators encountered in Taiwan. To be promoted from associate professors to professors, assistant professors to associate professors, and lecturers to assistant professors, the median equivalent number of papers calculated using the impact factor of the top-ranked journal and the lowest-ranked journal in primary health care required 4.6, 3.3, 2.5 and 30, 20, 13.5 papers, respectively.

If primary health care educators published numerous papers in low-impact factor journals, the limits on maximum paper counts in several medical schools in Taiwan would impede the achieving of target weighted scores by quantity. The impact factors of most journals ranking first in different specialties and subspecialties were higher than that of journals ranking first in primary health care. This implies that most impact factors from journals in different medical fields were higher than those from journals in family medicine. This could cause some family medicine researchers shift their goals and submit manuscripts to journals in other medical fields for reaching a higher impact factor or to obtain a higher weight from an impact factor itself instead of a ranking [21]. Another possible reason for not submitting manuscripts to journals in primary health care is that many family medicine researchers are devoted to epidemiology, public health, or other more suitable research fields [22]. Authorship order also influences career advancement, and the phenomenon of equal distribution designations becomes a trend in recent decades [23,24,25]. However, in a recent survey, nearly 40% of respondents considered this relatively evolving publication practice to be ethically questionable, therefore in need of policy guidance [24].

While most of the medical schools in Taiwan used a scoring system based on journal impact factors for the calculation of research impact, one medical school was an exception. CGU adopted a dual system wherein applicants could choose either total paper counts or the sum of SCI points for each paper. For applicants with publications in top journals, a single article may be sufficient to meet the criteria for the promotion. In this case, one publication in JAMA, with a 2019 journal impact factor of 45.54 would pass the threshold for promotion to professors. However, for those published in relatively low-impact factor or non-SCI journals such as domestic journals, increasing the number of published articles would be required to achieve the promotion. Even if family medicine educators published papers in the highest-ranked journal of primary health care (IF = 4.686), 16 such papers would be required to be promoted to professors. The promotion system in CGU seemingly encouraged the submission to top-tier journals and was not friendly toward family medicine research development.

Research output flourished in the past decades in Taiwan, and the Scientific Citation Index (SCI)- and impact factor (IF)-based performance assessments influenced the development of family medicine research [26]. Lin et al. investigated papers published by family medicine physicians in Taiwan and found that a lesser proportion of articles were published in the primary health care category and domestic SCIE journals compared to other countries [27]. Our study showed the weights from impact factors were important for achieving promotion thresholds; therefore, researchers tend to submit fewer manuscripts to journals with low weighted scores or domestic journals. In a cross-sectional analysis of universities worldwide, apart from impact factors, traditional academic promotion criteria also included authorship order and publications in faculties of biomedical sciences [9]. Recently, a survey conducted in universities of the United States and Canada revealed that publication quantity and journal name recognition were perceived as the most valued factors for promotion [28].

Though impact factors were mainly used in the scoring systems for academic promotion in Taiwan, an individual contribution to a certain academic field is not equivalent to impact factors [29]. Agarwal et al. also implied that publications in high-impact journals should not be the basis of selection for academic promotion or recruitment, as it would put junior researchers at a distinct disadvantage [30]. Researchers have advocated that the San Francisco Declaration on Research Assessment (DORA) principles and criteria for promotion, funding, and hiring should be reformed [31,32]. Other metrics have been used for academic assessment, including H-index, Eigen factor, and the newly proposed composite scores of six citation metrics [33,34]. Promotion committees are encouraged to employ a diverse range of parameters for judging promotions in research rather than assessments focused on a single metric [31,35].

In addition to the unfriendly promotion systems, one explanation for the impediment of academic promotion might be the lack of protected time for research. A national study in the US found that 38% of family medicine faculty spent no time on research, while another 42% spent one to four hours per week [36]. In a study conducted in medical schools in the US, low rates of promotion of generalists were observed in the intervening 17 years, which was due to a lower rate of publication [37]. Further study is required to establish the relation between time spent on research by family medicine faculty in Taiwan and the difference between their promotion rates as compared to other academic faculties.

This study also found non-SCIE/SSCI journals or domestic journals are taken as weighted scores in some medical schools, to promote local magazines and encourage teachers to publish. However, the weighted scores of these journals are relatively low or seldom considered in several medical schools. This finding is also in line with prior research that family medicine papers published in domestic journals had a disadvantage in terms of impact factors and readership [38]. Family medicine researchers might strive to publish their papers in international journals instead of domestic journals even though certain research topics were locally significant and suitable for domestic journals.

However, the present study also has limitations. First, an online search of websites was performed, and the data were restricted to the updated time, disregarding time gaps, if any, between the latest regulations of each medical school. Second, the impact factor would fluctuate every year, and the calculation of equivalents of papers might change over time. However, such changes are trivial and might not influence the core value of this study. Third, this work is based and relevant in Taiwan, but may not be representative of other geographic regions. Fourth, we compared the equivalent of papers in the top-most and lowest-ranked journals of primary health care; however, they only represent two extreme situations that are least likely to occur in real life. Fifth, publications of primary care researchers are not limited in the category of primary health care during academic promotion. Finally, we adopted an indirect method for family medicine educator’s academic promotion by investigating the scoring systems. Further research is required to directly explore the challenges faced by primary health care educators in achieving academic promotion in Taiwan. The actual impact of the scoring system on primary care physicians is worth investigating through interviews or other approaches.

## 5. Conclusions

This study investigated scoring systems of academic promotion in different medical schools in Taiwan. We compared the criteria for academic promotion of different specialists in these schools by calculating the required numbers of papers which was in turn influenced by impact factors. Academic promotion was incredibly challenging for primary health care educators in Taiwan. The relatively low-impact factors of primary health care journals resulted in more papers required for promotion. Nevertheless, the negative impacts of scoring systems require further investigation.

## Figures and Tables

**Table 1:** Family medicine teachers at the 13 medical schools in Taiwan (2019).

**Medical School**	Professor	Associate Professor	Assistant Professor	Lecturer	Total
National Taiwan University (NTU)	2	2	9	11	24
National Yang Ming Chiao Tung University (NYCU)	2	1	4	11	18
National Defense Medical Center (NDMC)		2	8	6	16
Taipei Medical University (TMU)		3	2	3	8
Fu Jen Catholic University (FJU)		2		4	6
Mackay Medical College (MMC)		2		3	5
Chang Gung University (CGU)			2		2
China Medical University (CMU)	4	1	4	3	12
Chung Shan Medical University (CSMU)	1	1			2
National Cheng Kung University (NCKU)	3	2			5
Kaohsiung Medical University (KMU)			4		4
I-Shou University (ISU)			1	3	4
Tzu Chi University (TCU)		3	2	5	10
Total	12	19	36	49	116

**Table 2:** Demonstration of the scoring system with examples from the medical school of National Yang Ming Chiao Tung University.

Item	Weighted Score
Category of papers (C)	
Original article	3 points
Short report article	2 points
Review article; limited to one per year	2 points
Case report	1 point
Impact factor (IF) or percentile ranking of SCI journals ^1^ (J)	
IF > 10	IF × 1.5
IF > 5 or Ranking ≤ 10%	8 points or IF
10% < Ranking ≤ 25%	6 points
25% < Ranking ≤ 50%	4 points
50% < Ranking ≤ 75%	3 points
Ranking lower than the 75%	2 points
Author order (A)	
First author or corresponding author	5.0 points
Second author	3.0 points
Third author	1.0 point
Fourth author or later	0.5 point

^1^ Percentile ranking of international Science Citation Index (SCI) journals is counted as the journal ranking divided by the total numbers of journals in the category; the newest version of InCites Journal Citation Reports (JCR) data shall prevail.

**Table 3:** Comparison of different weights conditional on an original article published by a clinician-educator as the first author in 13 medical schools in Taiwan.

Scoring System	NTU	NDMC	NYCU	TMU	FJU	MMC	CGU	CMU	CSMU	NCKU	KMU	ISU	TCU
Original article (C)	3	3	3	3	3	3		3	3	3	3	3	3
Ranking of an impact factor ^a^ (percentile) (J)													
Ranking < 5%	6	6	8	6	5	6		6	6	8	6	7.5	10
5% < Ranking ≤ 10%	6	6	8	6	5	6		6	6	6	6	7.5	8
Ranking > 80%	1	3	2	1	2	1		1	1	1	1	1	3
First author (A)	5	6	5	5	5	5		5	5	5	5	5	5

^a^ We only listed weights corresponding to journals with a rank ≤ 10% or >80% in any of the categories. NTU: National Taiwan University; NDMC: National Defense Medical Center; NYCU: National Yang Ming Chiao Tung University; TMU: Taipei Medical University; FJU: Fu Jen Catholic University; MMC: Mackay Medical College; CGU: Chang Gung University; CMU: China Medical University; CSMU: Chung Shan Medical University; NCKU: National Cheng Kung University; KMU: Kaohsiung Medical University; ISU: I-Shou University; TCU: Tzu Chi University.

**Table 4:** Comparison of the academic promotion scoring system based on the numbers of papers equivalent to different thresholds among 13 medical schools in Taiwan.

Equivalents of Papers	NTU	NDMC	NYCU	TMU	FJU	MMC	CGU ^a^	CMU	CSMU	NCKU	KMU	ISU	TCU
Equivalents of papers in the top ranked journal of primary health care (*)													
Professor	5.6 *	4.6 *	4.2	6.7	4.3	4.4	16	5.3	6.1	3.8	5.6	4	3.3
Associate professor	3.3 *	3.2 *	3.3	5	2.7	3.3	10.7	3.3	4.4	2.5	4.4	3.1	2
Assistant professor	2.8 *	2.3 *	2.5	3.3	2	2.8	5.3	2.3	2.8	1.9	3.3	1.6	1.3
Equivalents of papers in the lowest ranked journal of primary health care (*)													
Professor	33.3 *	9.3 *	16.7	40	10.7	26.7	35	31.5	36.7	30	33.3	30	16.7
Associate professor	20 *	6.5 *	13.3	30	6.7	20	20	20	26.7	20	26.7	23.3	10
Assistant professor	16.7 *	4.6 *	10	20	5	16.7	10	13.5	16.7	15.3	20	12	6.7

Note: * denote the number of papers in full score, with no threshold of a minimum requirement. ^a^ CGU employs a dual tract system either by paper counts or the sum of impact factors of each paper. NTU: National Taiwan University; NDMC: National Defense Medical Center; NYCU: National Yang Ming Chiao Tung University; TMU: Taipei Medical University; FJU: Fu Jen Catholic University; MMC: Mackay Medical College; CGU: Chang Gung University; CMU: China Medical University; CSMU: Chung Shan Medical University; NCKU: National Cheng Kung University; KMU: Kaohsiung Medical University; ISU: I-Shou University; TCU: Tzu Chi University.

**Table 5:** Maximum of papers counted for the promotion of different academic ranks in 13 medical schools in Taiwan.

Academic Rank	Maximum of Papers Counted					
	NTU	NYCU	TMU	MMC	NCKU	KMU
Professor	12	12	15	15	15	15
Associate professor	12	10	15	15	15	15
Assistant professor	12	8	15	15	15	15

NTU: National Taiwan University; NYCU: National Yang Ming Chiao Tung University; TMU: Taipei Medical University; MMC: Mackay Medical College; NCKU: National Cheng Kung University; KMU: Kaohsiung Medical University.

**Table 6:** Top-ranked journals of medical specialties and subspecialties.

Specialties	SCI Category	Top-Ranked Journal	
		Name	Impact Factor
Internal medicine	Medicine, General & Internal	New England Journal of Medicine	74.699
Gastroenterology	Gastroenterology & Hepatology	Nature Reviews Gastroenterology & Hepatology	29.848
Cardiology	Cardiac & Cardiovascular Systems	Circulation	23.603
Endocrinology & Metabolism	Endocrinology & Metabolism	Nature Reviews Endocrinology	28.8
Infectious Diseases	Infectious Diseases	Lancet Infectious Diseases	24.446
Nephrology	Urology & Nephrology ^1^	Nature Reviews Nephrology	20.711
Hematology	Hematology	Blood	17.543
Allergy, Immunology & Rheumatology	Allergy/ Immunology/Rheumatology	Nature Reviews Immunology	40.358
Chest Medicine	Respiratory System	Lancet Respiratory Medicine	25.094
Clinical Toxicology	Toxicology	Annual Review of Pharmacology and Toxicology	11.25
Surgery	Surgery	JAMA Surgery	13.625
Obstetrics and Gynecology	Obstetrics & Gynecology	Human Reproduction Update	12.684
Pediatrics	Pediatrics	JAMA Pediatrics	13.946
Family Medicine	Primary Health Care	Annals of Family Medicine	4.686
Ophthalmology	Ophthalmology	Progress in Retinal and Eye Research	14.86
Dermatology	Dermatology	Journal of the American Academy of Dermatology	8.277
Neurology	Clinical neurology	Lancet Neurology	30.039
Psychiatry	Psychiatry	World Psychiatry	40.595
Otorhinolaryngology	Otorhinolaryngology	JAMA Otolaryngology-Head & Neck Surgery	3.848
Radiology	Radiology, Nuclear Medicine & Medical Imaging	JACC-Cardiovascular Imaging	12.74
Urology	Urology & Nephrology ^2^	European Urology	17.947
Orthopedic Surgery	Orthopedics	American Journal of Sports Medicine	5.81
Nuclear Medicine	Radiology, Nuclear Medicine & Medical Imaging	JACC-Cardiovascular Imaging	12.74
Physical Medicine and Rehabilitation	Rehabilitation	Journal of Physiotherapy	5.44
Anesthesiology	Anesthesiology	Anesthesiology	7.067
Emergency Medicine	Emergency Medicine	Annals of Emergency Medicine	5.799
Pathology	Pathology	Annual Review of Pathology-Mechanisms of Disease	16.75
Geriatric Medicine	Geriatrics & Gerontology	Ageing Research Reviews	10.616
Environmental and Occupational Medicine	Public, Environmental, & Occupational Health	Lancet Global Health	21.597

^1^ In the 2019 Edition of InCites Journal Citation Reports, nephrology has been classified in the subject category of “Urology & Nephrology”. Here, we only considered the journals related to nephrology. ^2^ In the 2019 Edition of InCites Journal Citation Reports, urology has been classified in the subject category of “Urology & Nephrology”. Here, we only considered the journals related to urology.

## Data Availability

Data is contained within the article and supplementary materials.

## Supplementary Materials

The following are available online at https://www.mdpi.com/article/10.3390/ijerph18189615/s1, Table S1: Websites of academic promotion regulations of 13 medical schools in Taiwan.

## References

[1] Greenberg L., Bickel J. (2010). Teaching scholarship and the clinician/educator. Pediatr. Ann..

[2] Sherbino J., Frank J.R., Snell L. (2014). Defining the key roles and competencies of the clinician-educator of the 21st century: A national mixed-methods study. Acad. Med..

[3] Chapman T., Carrico C., Vagal A.S., Paladin A.M. (2012). Promotion as a clinician educator in academic radiology departments: Guidelines at three major institutions. Acad. Radiol..

[4] Pilcher E.S., Kilpatrick A.O., Segars J. (2009). An assessment of promotion and tenure requirements at dental schools. J. Dent. Educ..

[5] Hunt R.J., Gray C.F. (2002). Faculty appointment policies and tracks in U.S. dental schools with clinical or research emphases. J. Dent. Educ..

[6] Carter R.E. (1992). Criteria for the academic promotion of medical school-based psychiatrists. Acad. Psychiatry.

[7] Ence A.K., Cope S.R., Holliday E.B., Somerson J.S. (2016). Publication productivity and experience: Factors associated with academic rank among orthopaedic surgery faculty in the United States. J. Bone Jt. Surg. Am..

[8] Jacobs C.K., Everard K.M., Cronholm P.F. (2020). Promotion of clinical educators: A critical need in academic family medicine. Fam. Med..

[9] Rice D.B., Raffoul H., Ioannidis J.P.A., Moher D. (2020). Academic criteria for promotion and tenure in biomedical sciences faculties: Cross sectional analysis of international sample of universities. BMJ.

[10] Sox H.C., Schuster M.A. (2020). Criteria for academic promotion in medicine. BMJ.

[11] (2019). In Cites Journal Citation Reports. https://jcr.clarivate.com/jcr/home.

[12] Population by Sex and 5 Year Age Group for Counties and Cities of Taiwan. https://www.ris.gov.tw/app/en/3910.

[13] Hou Y.C., Oren G.A., Chen M.S., Hu F.R. (2016). History and development of ophthalmology in Taiwan. J. Formos. Med. Assoc..

[14] Medical Statistics-Taiwan Medical Association. https://www.tma.tw/stats/index_NYearInfo.asp?/2019.html.

[15] Chu T.S., Weed H.G., Yang P.C. (2009). Recommendations for medical education in Taiwan. J. Formos. Med. Assoc..

[16] Chen S.H., Chang H.T., Lin M.H., Chen T.J., Hwang S.J., Lin M.N. The status quo of family medicine workforce at medical schools in Taiwan. Proceedings of the Taiwan Association of Family Medicine 2020 Annual Academic Seminar, National Taiwan University Hospital.

[17] Act Governing the Appointment of Educators. https://edu.law.moe.gov.tw/EngLawContent.aspx?lan=E&id=178.

[18] Academics, National Yang Ming Chiao Tung University, School of Medicine. https://md-e.ym.edu.tw/files/11-1242-4.php.

[19] Standards for Assessment and Accreditation of Internal Medicine Programs. http://www.tsim.org.tw/trn/內科認定基準OK_1050719.pdf.

[20] The R Project for Statistical Computing. https://www.r-project.org/.

[21] Sebo P. (2020). General internal medicine and family medicine journals: Comparative study of published articles using bibliometric data. Medicine.

[22] Pshetizky Y., Tandeter H., Tabenkin H., Vinker S., Lahad A., Karkabi K., Kitai E., Hermoni D., Shvartzman P. (2009). Thirty years of family medicine publications in Israel (1975−2004): What, where, and how much?. J. Am. Board Fam. Med..

[23] Aguzzi A. (2021). Equal contribution means that the contribution is equal. Swiss Med. Wkly..

[24] Resnik D.B., Smith E., Master Z., Shi M. (2020). Survey of equal contributions in biomedical research publications. Acc. Res..

[25] Li Z., Sun Y.M., Wu F.X., Yang L.Q., Lu Z.J., Yu W.F. (2013). Equal contributions and credit: An emerging trend in the characterization of authorship in major anaesthesia journals during a 10-yr period. PLoS ONE.

[26] Lin M.H., Chen L.K., Hwang S.J., Weiss B.D., Chou L.F., Chen T.J. (2006). The impact of impact factor on small specialties: A case study of family medicine in Taiwan. Scientometrics.

[27] Lin M.H., Hwang S.J., Hwang I.H., Chen Y.C. (2014). Family medicine publications in Taiwan: An analysis of the Web of Science database from 1993 to 2012. J. Chin. Med. Assoc..

[28] Niles M.T., Schimanski L.A., McKiernan E.C., Alperin J.P. (2020). Why we publish where we do: Faculty publishing values and their relationship to review, promotion and tenure expectations. PLoS ONE.

[29] Russell R., Singh D. (2009). Impact factor and its role in academic promotion. Int. J. Chron. Obstruct. Pulm. Dis..

[30] Agarwal A., Durairajanayagam D., Tatagari S., Esteves S.C., Harlev A., Henkel R., Roychoudhury S., Homa S., Puchalt N.G., Ramasamy R. (2016). Bibliometrics: Tracking research impact by selecting the appropriate metrics. Asian J. Androl..

[31] Casadevall A., Fang F.C. (2014). Causes for the persistence of impact factor mania. mBio.

[32] Schmid S.L. (2017). Five years post-DORA: Promoting best practices for research assessment. Mol. Biol. Cell.

[33] Ioannidis J.P.A., Baas J., Klavans R., Boyack K.W. (2019). A standardized citation metrics author database annotated for scientific field. PLoS Biol..

[34] Chapman C.A., Bicca-Marques J.C., Calvignac-Spencer S., Fan P., Fashing P.J., Gogarten J., Guo S., Hemingway C.A., Leendertz F., Li B. (2019). Games academics play and their consequences: How authorship, H-index and journal impact factors are shaping the future of academia. Proc. Biol. Sci..

[35] Moher D., Naudet F., Cristea I.A., Miedema F., Ioannidis J.P.A., Goodman S.N. (2018). Assessing scientists for hiring, promotion, and tenure. PLoS Biol..

[36] Brocato J.J., Mavis B. (2005). The research productivity of faculty in family medicine departments at U.S. medical schools: A national study. Acad. Med..

[37] Blazey-Martin D., Carr P.L., Terrin N., Breeze J.L., Luk C., Raj A., Freund K.M. (2017). Lower rates of promotion of generalists in academic medicine: A follow-up to the National faculty survey. J. Gen. Intern. Med..

[38] Peleg R., Shvartzman P. (2006). Where should family medicine papers be published—Following the impact factor?. J. Am. Board Fam. Med..

