# Supplementary material for: Academic Promotion of Physicians in Medical Schools: A Special Focus on Primary Health Care in Taiwan
Source: Int J Environ Res Public Health. 2021 Sep 12;18(18):9615. doi: 10.3390/ijerph18189615 (PMC8465314; doi:10.3390/ijerph18189615)
Supplement: Supplementary file 1 [file ijerph-18-09615-s001.zip › ijerph-1367710-supplementary.pdf]

International Journal of
Environmental Research and Public Health

Supplementary Material

Table S1. Websites of academic promotion regulations of 13 medical schools in Taiwan (accessed on 21 March 2021).

Medical school	Websites
NTU	https://www.mc.ntu.edu.tw/person/Fpage.action?muid=2041&fid=1368
NYCU	https://som.ym.edu.tw/files/11-1153-41.php
NDMC	https://wwwndmc.ndmctsgh.edu.tw/unit/100003/1114
TMU	http://tmu-hr.tmu.edu.tw/zh_tw/pl1
FJU	http://www.med.fju.edu.tw/DepIntro.jsp?DI_CODE=5
MMC	http://www.personnel.mmc.edu.tw/TechEval.asp
CGU	https://personnel.cgu.edu.tw/p/404-1005-14870.php
CMU	https://regulation.cmu.edu.tw/statute_list.php?class=2&dept=28
CSMU	http://msg.csmu.edu.tw/message/MSG004.aspx?admin=7
NCKU	https://www.cc.ncku.edu.tw/rule/detail.php?DEPNO1=74000000
KMU	https://cmed.kmu.edu.tw/index.php/zh-TW/升等-新聘教師專區/升等教師/353-109-2專-兼任「升等」教師【著作送審】專區
ISU	https://www.isu.edu.tw/2018/show-page_v01.php?dept_mno=633&dept_id=7&page_id=33784
TCU	https://law.tcu.edu.tw/Law?deptCode=310100

NTU: National Taiwan University; NYCU: National Yang Ming Chiao Tung University; NDMC: National Defense Medical Center; TMU: Taipei Medical University; FJU: Fu Jen Catholic University; MMC: Mackay Medical College; CGU: Chang Gung University; CMU: China Medical University; CSMU: Chung Shan Medical University; NCKU: National Cheng Kung University; KMU: Kaohsiung Medical University; ISU: I-Shou University; TCU: Tzu Chi University.
